# Long-term stimulation with alternating electric fields modulates the differentiation and mineralization of human pre-osteoblasts

**DOI:** 10.3389/fphys.2022.965181

**Published:** 2022-09-30

**Authors:** Franziska Sahm, Vivica Freiin Grote, Julius Zimmermann, Fiete Haack, Adelinde M. Uhrmacher, Ursula van Rienen, Rainer Bader, Rainer Detsch, Anika Jonitz-Heincke

**Affiliations:** ^1^ Biomechanics and Implant Technology Research Laboratory, Department of Orthopedics, Rostock University Medical Centre, Rostock, Germany; ^2^ Chair of Theoretical Electrical Engineering, Institute for General Electrical Engineering, University of Rostock, Rostock, Germany; ^3^ Institute for Visual and Analytic Computing, University of Rostock, Rostock, Germany; ^4^ Department Life, Light and Matter, University of Rostock, Rostock, Germany; ^5^ Department Ageing of Individuals and Society, University of Rostock, Rostock, Germany; ^6^ Department of Materials Science and Engineering, Institute of Biomaterials, Friedrich Alexander-University Erlangen-Nuremberg, Erlangen, Germany

**Keywords:** pre-osteoblasts, differentiation, electric stimulation, alternating fields, long-term stimulation, bone remodeling

## Abstract

Biophysical stimulation by electric fields can promote bone formation in bone defects of critical size. Even though, long-term effects of alternating electric fields on the differentiation of osteoblasts are not fully understood. Human pre-osteoblasts were stimulated over 31 days to gain more information about these cellular processes. An alternating electric field with 0.7 V_rms_ and 20 Hz at two distances was applied and viability, mineralization, gene expression, and protein release of differentiation factors were analyzed. The viability was enhanced during the first days of stimulation. A higher electric field resulted in upregulation of typical osteogenic markers like osteoprotegerin, osteopontin, and interleukin-6, but no significant changes in mineralization. Upregulation of the osteogenic markers could be detected with a lower electric field after the first days of stimulation. As a significant increase in the mineralized matrix was identified, an enhanced osteogenesis due to low alternating electric fields can be assumed.

## 1 Introduction

Understanding bone remodeling processes becomes more important as the global population is getting older. The percentage of the world population aged over 65 years is presumed to rise from 14.3% in 2020 to 25.3% in 2050 to 33% in 2080, more than doubling within the next 60 years (United Nations, Department of Economic and Social Affairs, 2019). Hence, characteristics of aging will affect the quality of their life. Typical problems during aging, such as the wear and tear of joints, can lead to limited physical mobility. The reduction of bone density and impaired healing capacities result in a higher risk of bone fractures and hip arthroplasty (Maier et al., 2016; Carvalho et al., 2021). Thus, unharmed bone healing and remodeling processes are essential for the successful regeneration and osseointegration of artificial joints (Gruber et al., 2006).

Currently, severe complications occur while healing in 5–10% of bone fractures and 1–5% of joint replacements with a revision rate of less than 5% beyond 10 years for total hip replacements (Crawford and Murray, 1997; Buza and Einhorn, 2016; Khan et al., 2016; Ferguson et al., 2018). One possibility to enhance osseointegration and to increase the early and stable fixation of bone implants are biophysical and mechanotransduction processes, particularly the stimulation with exogenous electric fields (Bhavsar et al., 2020; Hao et al., 2021). After applying mechanical loading, Fukada and Yasuda discovered endogenous electric fields in bone (Fukada and Yasuda, 1957). The described piezoelectricity can be reversed. It is already successfully used in different electrical stimulation systems for bone non-unions, ankle and foot unions, spinal fusions, and necrosis of the femoral head (Griffin and Bayat, 2011; Ellenrieder et al., 2013; Bhavsar et al., 2020).

Several studies were conducted *in vitro* to understand the underlying processes. At the cellular level, the exogenously generated electric fields induce various electrocoupling mechanisms that cause asymmetric redistribution or diffusion of electrically charged molecules on the cell membrane, activating numerous downstream signaling cascades (Balint et al., 2012; Chen et al., 2019). Another effector mechanism may be related to cell membrane depolarization through direct activation of voltage-gated Ca^2+^ ion channels (Babona-Pilipos et al., 2018; Leppik et al., 2020). In addition, the inverse piezoelectric effect is widely discussed: an electrical stimulus leads to mechanical strain, resulting in either direct reorganization of cytoskeletal filaments or interfering with cellular processes regulated by the cytoskeleton (Leppik et al., 2020). Recent *in vitro* studies on electrical stimulation demonstrate the pro-healing potential of osseous cells following electrical stimulation. In general, cell behavior can be influenced with respect to migration, proliferation, differentiation, formation of extracellular matrix, and mineralization (Leppik et al., 2020). For osteoblastic cells, the influence on phenotype expression and differentiation factors, like alkaline phosphatase (ALP), collagen type 1 and calcium deposition, could be demonstrated (Ercan and Webster, 2010; dos Santos et al., 2016; Portan et al., 2019). These findings were obtained using a variety of different test systems, including approaches for capacitive, inductive, magnetic, or direct coupling of the electric fields (Thrivikraman et al., 2018; Leppik et al., 2020). However, most studies focus on pulsed electromagnetic fields, whereas only a few studies exist on direct coupling (deVet et al., 2021). Furthermore, mainly direct current signals are used, accompanied by significant side effects due to electrochemical reactions on the electrode (Thrivikraman et al., 2018). The application of alternating fields can prevent such chemical reactions and further could act as a pump to move ions and waste to and from cells in the absence of vessels. (Balint et al., 2012; deVet et al., 2021).

The stimulation system used for direct coupling of alternating electric fields is based on the ASNIS IIIs screw system. The clinically used electrode system served as the basis for designing an electrode which can be used for *in vitro* studies but still has similarities to the *in situ* used stimulation device (Hiemer et al., 2018). This should help to improve the applicability of *in vitro* gained results. As studies mainly focused on short-term stimulation up to 7–14 days, current research focuses on the influence of long-term stimulation on the differentiation and mineralization behavior of osteoblastic cells up to 31 days (Sahm et al., 2020; deVet et al., 2021). The present study aimed to clarify how the initial induction of osteogenic differentiation can be maintained over a more extended stimulation period using the direct stimulation device. As *in vivo* studies and clinical applications often last longer than 14 days, the present study extended investigation of cell effects up to 31 days. In this context, the study by McCullen et al. (2010) already proves that prolonged stimulation over 14 days increases the mineralization capacity and the release of calcium in adipogenic stem cells and thus osteogenic differentiation (McCullen et al., 2010). de Sousa et al. (2021) analyzed the protein synthesis of osteonectin and collagen type 1 of osteoblasts under the influence of high frequencies using a capacitive coupled system over 28 days and revealed significant changes at different time points (de Sousa et al., 2021). With the following study a broader spectrum of differentiation factors and signaling molecules should be analyzed using a direct stimulation system with low alternating electrical fields. A more detailed observation of the mineralization processes was done to receive further information about the calcium deposition under electric stimulation over time. To reveal new signaling cascades which might be influenced through the electric stimulation, a transcriptome analyses of cells stimulated over 7 and 28 days was performed.

## 2 Materials and methods

### 2.1 Isolation and cultivation of human primary pre-osteoblasts

Human primary pre-osteoblasts were isolated from patients undergoing total hip replacement as described previously (Lochner et al., 2011). Femoral heads were collected under sterile conditions with the patients’ consent, following approval by the Local Ethical Committee (Registration number: A 2010-0010, approval date: 27 January 2017). In brief, the spongiosa was isolated and digested with collagenase a and dispase (both: Roche, Basel, Switzerland). The cell suspension was filtered and centrifuged for further purification. Afterward, it was transferred in cell culture flask and cultivated in Dulbecco’s Modified Eagle Medium (DMEM, PAN-Biotech, Aidenbach, Germany) without calcium, containing 10% fetal calf serum (FCS, PAN-Biotech, Aidenbach, Germany), 1% amphotericin B, 1% penicillin-streptomycin, and 1% HEPES buffer (all: Sigma-Aldrich, Munich, Germany). CaCl_2_ was reduced to enhance proliferation and to maintain the immature stage of the osteoblasts. Ascorbic acid (final concentration: 50 μg/mL), β-glycerophosphate (final concentration: 10 mM), and dexamethasone (final concentration: 100 nM) (all: Sigma-Aldrich, Munich, Germany) were added to the cell culture medium to prevent cells from dedifferentiation and promoting the osteogenic stage of the cells (Coelho and Fernandes, 2000). All cultivation steps were performed under standard cell culture conditions (5% CO_2_ and 37°C). After two passages, ALP activity was tested on a random basis to ensure the pre-osteoblastic cell stage. The cells were stored in liquid nitrogen until usage.

For the stimulation experiments, pre-osteoblasts from a total of 19 different donors, ten females (age: 72.3 ± 8.68 years) and nine males (age: 73.3 ± 6.3 years) were thawed and cultured for another passage using the same medium and osteogenic additives (ascorbic acid, β-glycerophosphate, dexamethasone) as described above. 30,000 cells, each from a different donor, were seeded on a rat tail collagen-coated coverslip (diameter: 15 mm, Neuvitro Corporation, Vancouver, WA, United States) placed in the center of a 6-well plate. After adhering for 30 min at room temperature, 5 mL of cell culture medium was added. The medium contained the osteogenic additives with the addition of 200 mg/L CaCl_2_ for activating mineralization processes and promote further cell differentiation into mature osteoblasts during stimulation (Coelho and Fernandes, 2000). This medium composition was used for all experiments.

### 2.2 Electrical stimulation protocol

An *in vitro* setup for a 6-well cell culture plate, developed earlier by our working group, was used to analyze the influence of electrical stimulation on pre-osteoblasts (Hiemer et al., 2018). The stimulation system is based on the clinically used ASNIS IIIs screw system, which is a semi-invasive bone formation stimulating implant. In this system, two electrodes separated by an insulator are integrated into a screw that can be implanted into the femoral head. The system can be used to apply electromagnetic fields with an additional alternating electric field between 5 and 70 V/m, and its operation is based on the bipolar induction screw system (BISS) (Mittelmeier et al., 2004). The electric field delivered with the stimulation parameters is expected to stimulate the peri-implant bone tissue and thus accelerate bone regeneration (Grunert et al., 2014; Su et al., 2014). This electrode arrangement was appropriately adapted for cell culture to induce the alternating electric fields directly without the use of a magnetic coil. The miniaturization further allows a reduction in the volume of medium used, which can significantly increase the concentration of secreted proteins for further protein analysis.

**FIGURE 1:**
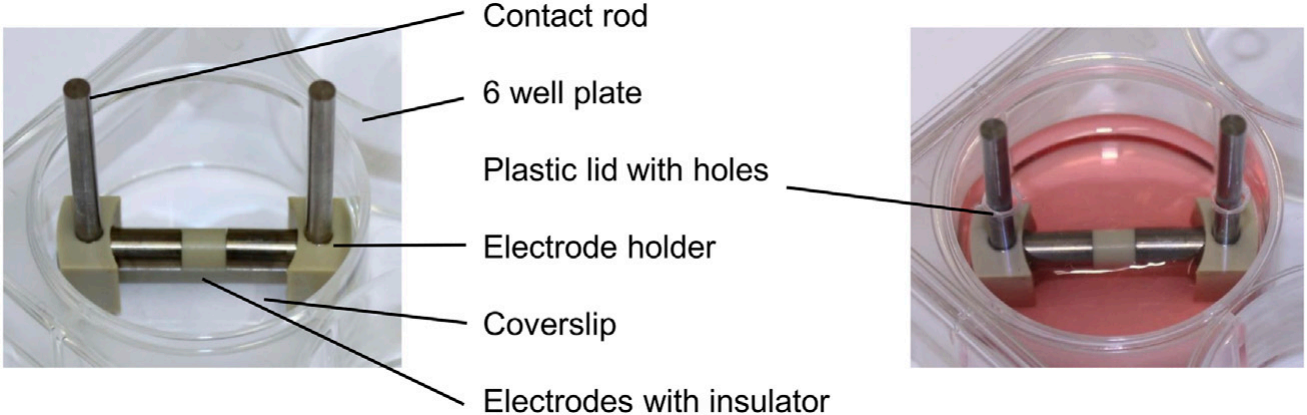
Electrical stimulation device without and with culture medium in a 6-well plate consisting of contact rods, electrode holders, and the electrodes connected over an insulator.

Each electrode for the direct electrical stimulation comprises two Ti6Al4V cylindrical electrodes, separated by a 5 mm long insulator made of polyetheretherketone (PEEK) (Figure 1). The electrode holders were made of PEEK and can generate a 1 mm or 3 mm gap between the electrodes and the coverslips positioned on the well bottom. Thus, it is possible to generate two different electric fields: a higher electric field with the 1 mm gap and a lower one with a 3 mm gap. Voltage was applied over the Ti6Al4V contact rods using a Metrix GX 305 and GX 310 function generator (Metrix Electronics, Bramley, Hampshire, United Kingdom). The electrical stimulation started 24 h after cell seeding. A sinusoidal signal with 0.7 V_rms_ and a frequency of 20 Hz was used. The 1 mm and the 3 mm gaps were used to mimic the periprosthetic gap between the electrode and the surrounding tissue *in vivo*. The AC voltage was applied three times a day for 45 min with 225 min breaks between stimulations and a longer 855 min break. Electrical stimulation was done using the two different gaps, and unconnected electrodes were used for the unstimulated controls. For each time point and assay, the matching unstimulated control, cultured the same amount of time as the stimulated cells, served as a control. Gene expression data were generated from day 1 to day 28 to gather information about changes in transcription directly after the stimulation was started. The mineralization was analyzed from day 3 to day 31 as mineralization processes are only detectable at later time points. The metabolic activity and the protein release in the medium were observed at each time point. Samples were taken always 20 h after the last stimulation interval was started. All cultivation steps were implemented using standard cell culture conditions as mentioned above. The medium was exchanged every 7 days.

### 2.3 Electric field simulation

Two distances between the electrode and the well bottom were used to generate two different electric fields. The small distance of 1 mm led to a higher electric field (HEF) and the 3 mm to a lower electric field (LEF). Numerical simulations were performed to get a first idea of the magnitudes of the LEF and HEF. The electric field strength was computed using the Finite Element Method (FEM). Laplace’s equation was solved on the cell culture medium domain. The height of the cell culture medium in the center of the well was estimated by visual inspection. In this work, we did not consider the meniscus arising due to capillary effects at the well’s walls and the electrode holder. The numerical solution was post-processed to obtain the total current through the medium. Convergence was ensured by locally refining the mesh and applying adaptive mesh refinement. More detailed explanations of the underlying theoretical and numerical approach can be found in an earlier publication (Zimmermann J et al., 2021). The simulations were performed using NGSolve (Schöberl et al., 2014) and the Netgen mesh generator (Schöberl, 1997). COMSOL Multiphysics was employed to verify the correctness of the results.

### 2.4 Cell viability

The cell viability was determined after 1, 3, 7, 14, 21, 28, and 31 days of stimulation, each 20 h after the last stimulation interval started. The coverslips with the cells were transferred from the 6-well plate to a 12-well plate. The water-soluble tetrazolium salt (WST-1) assay (Takara, Gothenburg, Sweden) was used in a ratio of 1:10 with DMEM and transfused on the cells. The reagent was incubated over 45 min at 37°C and 5% CO_2_. 100 µL of the solution were transferred as duplicates into a 96-well plate. The color change was quantified using the multimode plate reader Infinite 200 pro (Tecan Group Ltd., Maennedorf, Switzerland) at a wavelength of 450 nm and a reference filter of 630 nm. The WST-1 and DMEM solution blank was carried along with each series and subtracted from the measured values.

### 2.5 Assessment of alkaline phosphatase activity

The activity of the intracellularly generated ALP was analyzed after 1, 3, 7, 14, 21, and 28 days of stimulation, each 20 h after the last stimulation interval started. The cells were washed twice using TRIS buffer (50 mM, pH = 8.0) lysed with 1% Triton X and 1% phenylmethylsulfonyl fluoride (both: Merck, Darmstadt, Germany) for 10 min. A solution containing 10 mM 4-Nitrophenylphosphat (AppliChem, Darmstadt, Germany), 100 mM 2-amino-2-methyl-1,3-propanediol (Sigma-Aldrich, Munich, Germany), and 5 mM magnesium chloride (Merck, Darmstadt, Germany) was added to the lysate and incubated over 1 h at 37°C and 5% CO_2_. The reaction was stopped with a 2 M sodium hydroxide solution, and the absorption was measured at 405 nm with multimode plate reader Infinite 200 pro (Tecan Group Ltd., Maennedorf, Switzerland). A blank served as an internal control and was subtracted from each value.

### 2.6 Gene expression analysis

Gene expression was analyzed after 1, 3, 7, 14, 21, and 28 days of stimulation, each 20 h after the last stimulation interval started. Coverslips with the cells were transferred from a 6-well to a 12-well plate and lysed with the peqGOLD Total RNA Kit (VWR International GmbH, Darmstadt, Germany), following the manufacturer’s instructions, to analyze the expression of genes associated with osteogenic differentiation. The purified RNA was eluted with 25 µL of sterile RNase-free water (Carl Roth GmbH & Co. KG, Karlsruhe, Germany) and RNA concentration was determined using the plate reader Infinite 200 pro. The High Capacity cDNA Reverse Transcription Kit (Thermo Fisher Scientific, Waltham, MA, United States) was used for the transcription of 100 ng RNA into complementary DNA (cDNA) following the manufacturer’s instructions. The program was run at 25°C for 10 min, 37°C for 120 min and 85°C for 15 s. The cDNA was diluted 1:1 with nuclease free water, and frozen at −20°C until further usage. Samples were thawed on ice for the semi-quantitative reverse transcription-polymerase chain reaction (qPCR). The PCR was done in duplicates using the innuMIX qPCR MasterMix SyGreen Kit (Analytik Jena, Jena, Germany). The samples were heated up to 95°C for 2 min, and a cycle of 40 reruns was processed with 95°C for 5 s and 60–65°C for 25 s. Primers for the genes of interest are listed in Table 1. The delta-delta Ct (∆∆Ct) method was used to evaluate the results (Livak and Schmittgen, 2001). HPRT was used as a housekeeper gene, and the simulation samples were related to the matching control.

**TABLE 1:** Primer sequences for the genes of interest.

Gene	Sequence
Alkaline phosphatase (ALPL)	For: 5′-CAT​TGT​GAC​CAC​CAC​GAG​AG-3′
	Rev: 5′-CCA​TGA​TCA​CGT​CAA​TGT​CC-3′
Alpha-1 type I collagen (COL1A1)	For: 5′-ACG​AAG​ACA​TCC​CAC​CAA​TC-3′
	Rev: 5′-AGA​TCA​CGT​CAT​CGC​ACA​AC-3′
Caspase 8 (CASP8)	For: 5′-TGT​TTT​CAC​AGG​TTC​TCC​TCC​TTT-3′
	Rev: 5′-GAG​AAT​ATA​ATC​CGC​TCC​ACC​TT-3′
Hypoxanthine-guanine phosphoribosyl transferase (HPRT)	For: 5′-CCC​TGG​CGT​CGT​GAT​TAG​TG-3′
	Rev: 5′-TCG​AGC​AAG​ACG​TTC​AGT​CC-3′
Integrin binding sialoprotein (IBSP)	For: 5′- ATT​TTG​GGA​ATG​GCC​TGT​GC-3′
	Rev: 5′- GTC​ACT​ACT​GCC​CTG​AAC​TGG-3′
Bone gamma-carboxyglutamate protein—osteocalcin (BGLAP)	For: 5′-TCA​GCC​AAC​TCG​TCA​CAG​TC-3′
	Rev: 5′-GGT​GCA​GCC​TTT​GTG​TCC-3′
Secreted protein acidic and cysteine rich-osteonectin (SPARC)	For: 5′- CTG​GAC​TAC​ATC​GGG​CCT​TG-3′
	Rev: 5′- ATG​GAT​CTT​CTT​CAC​CCG​CAG-3′
Secreted phosphoprotein 1—osteopontin (SPP1)	For: 5′-AAC​GCC​GAC​CAA​GGA​AAA​CT-3′
	Rev: 5′-GCA​CAG​GTG​ATG​CCT​AGG​AG-3′
Receptor activator of nuclear factor-kappa-Β ligand (RANKL)	For: 5′-TCT​TCT​ATT​TCA​GAG​CGC​AGA​TGG-3′
	Rev: 5′-CTG​ATG​TGC​TGT​GAT​CCA​ACG-3′
Runt-related transcription factor 2 (RUNX2)	For: 5′- CGC​CTC​ACA​AAC​AAC​CAC​AG-3′
	Rev: 5′- ACT​GCT​TGC​AGC​CTT​AAA​TGA​C-3′

### 2.7 Quantification of the secreted proteins

The supernatants used for the quantification of secreted proteins were collected after 1, 3, 7, 14, 21, 28, and 31 days of stimulation, each 20 h after the last stimulation interval started.

#### 2.7.1 Quantification of secreted procollagen type I and osteopontin

The type I C-terminal collagen pro peptide (CICP), and osteopontin were used as markers for the differentiation capacity of the pre-osteoblasts. The supernatants containing CICP and osteopontin were analyzed using enzyme-linked immunosorbent assays (ELISA). For CICP, the MicroVue CICP ELISA (Quidel, San Diego, CA, United States), and for osteopontin, the Human Osteopontin SimpleStep ELISA Kit (Abcam, Cambridge, United Kingdom) were used. The analyses were done following the manufacturer’s instructions, and internal standards served to determine the concentration of each protein. The absorption was measured using a microplate reader (Tecan Trading AG, Maennedorf, Switzerland) at a wavelength of 405 nm. The measured protein concentration was normalized to the total protein content of each supernatant. For this purpose, the Invitrogen Qubit Protein Assay Kit and the Qubit fluorometer Q32857 (both: Thermo Fisher Scientific, Waltham, MA, United States) were used according to the manufacturer’s instructions. Included standards were used to quantify the total protein content.

#### 2.7.2 Quantification of the secreted interleukin-6, dickkopf-related protein 1 and osteoprotegerin

Secreted interleukin-6 (IL-6), dickkopf-related protein 1 (DKK-1), and osteoprotegerin (OPG) were analyzed in the supernatant of each sample with a customized human BioLegend’s LEGENDplex™ multiplex assay (Biolegend, San Diego, CA, United States) containing antibodies for IL-6, DKK-1, and OPG. Analysis was done following the manufacturer’s instructions, and internal standards served to determine the concentration of each protein. The multiplex assay was measured with a BD FACSVerse™ (Becton, Dickinson and Company, Franklin Lakes, NJ, United States) and analyzed with the LEGENDplex™ Data Analysis Software. The measured protein concentration was normalized to each supernatant’s total protein content (see 2.7.1).

### 2.8 Quantification of mineralization

The amount of calcium phosphate mineralization was determined after 1, 3, 7, 14, 21, 28, and 31 days of stimulation, each 20 h after the last stimulation interval started. The mineralization processes were examined on top of the cells and the surrounding well bottom. The glass coverslips were transferred to a 12-well plate to analyze the amount of calcium nodules on the cell layer. They were washed with PBS, fixed with PFA for 10 min (Grimm med. Logistik GmbH, Torgelow, Germany), and washed with deionized water before staining with 1% alizarin red (Santa Cruz Biotechnology, Dallas, TX, United States). After an incubation of 10 min, the coverslips containing the cell monolayer were rewashed with deionized water to remove the excess dye. The glass coverslips were dried at room temperature overnight. Pictures of the entire coverslip were generated using the digital microscope VHX-6000 (Keyence, Osaka, Japan) with an automatically stitching process of single pictures taken with a 200x magnification. The percentage of the colored surface area of the entire coverslip was determined with the open-source software ImageJ by three different researchers, and the mean value was used to prevent subjective evaluation.

The amount of deposited calcium surrounding the cell-seeded coverslips was analyzed using the remaining 6-well plates (Supplementary Figure S1). After removing the coverslips containing the cells, the well’s bottom was washed with deionized water, and the calcium layer was dissolved by adding 2 ml of 0.5 M HCl. After overnight incubation, the pH was neutralized using 2 M NaOH. The calcium concentration was measured with the colorimetric Calcium Assay Kit (Abcam, Cambridge, United Kingdom), and internal standards served to determine the concentration. Measurements without human pre-osteoblast served further as controls for calcium deposition in the LEF or in unstimulated wells (Supplementary Figure S2). The assay was carried out following manufactures instructions, and the measurement was done at a wavelength of 575 nm using a microplate reader (Tecan Trading AG, Maennedorf, Switzerland).

### 2.9 Transcriptome analysis

The transcriptome analysis was performed by ATLAS Biolabs (Berlin, Germany). Pre-osteoblasts from three different donors were stimulated in duplicates 7 and 28 days with the HEF and without electric stimulation (control). The total RNA of each donor was isolated and afterward pooled from the three donors. The pooling was necessary due to the need of a high RNA amount for the transcriptome analysis and the comparatively low cell number used in the experiments. In the transcriptome analysis data set provided by ATLAS Biolabs, the signal intensity of more than 55,335 annotated probe sets, hence RNA transcripts were determined.

Based on the normalized, logarithmic (basis 2) probe set intensity measured for each transcript in pooled control and HEF stimulated cells, fold change values were calculated for each stimulation time, i.e., for cells stimulated 7 or 28 days.

For pathway analysis the data set was further reduced by removing all transcripts with missing gene description or any GO-annotation referring to biological or molecular functions. Further we concentrated on genes and omitted functional RNA species, such as miRNA.

The pathway enrichment analysis was done using the g:profiler and EnrichmentMap pipeline, as described in Reimand et al. (2019) (Reimand et al., 2019). The version of g:profiler used in this analysis was e105_eg52_p16_e84549f, with database update on 03/01/2022. Only annotated genes were used in the analysis and all queries were issued with the following parameters. The organism h. sapiens was chosen. As data sources, molecular function and biological process of gene ontology (GO) were used as annotations, and KEGG and Reactome were used as pathway data bases. The resulting data annotation set and gene enrichment map (gmt and gem files, respectively) were downloaded and subsequently used for visualization in cytoscape version 3.9.1. (Shannon et al., 2003).

### 2.10 Display of the data and statistical analysis

The data obtained in this study were depicted related to the unstimulated control (100%) of each time point. Therefore, every graph shows the changes resulting from the electric stimulations compared to the related unstimulated control. The data were shown in heatmaps and individual values with median and the 25%- and 75%-quartile. The heatmaps show the median while an upregulation with a median higher than the related control is shown in blue, a downregulation with a lower median is shown in orange.

The data were statistically analyzed using GraphPad Prism software (GraphPad Software, San Diego, CA, United States). For all experiments, a minimum of five replicates, each from a different donor, were used for each time point. The normal distribution was verified using the Shapiro-Wilk test. The results of each electrical stimulation and their respective control were compared with a paired *t*-test for normal distribution or a Wilcoxon for not normal distribution to identify significances. Differences resulting from stimulation time were analyzed using a one-way ANOVA with Tukey for normal distribution or a Kruskal-Wallis Test with Dunn’s for not normal distribution. The results for the two different electric fields were compared with a two-way ANOVA, but no significant changes (*p* < 0.05) could be determined.

## 3 Results

### 3.1 Numerical simulation of alternating electric fields

**FIGURE 2:**
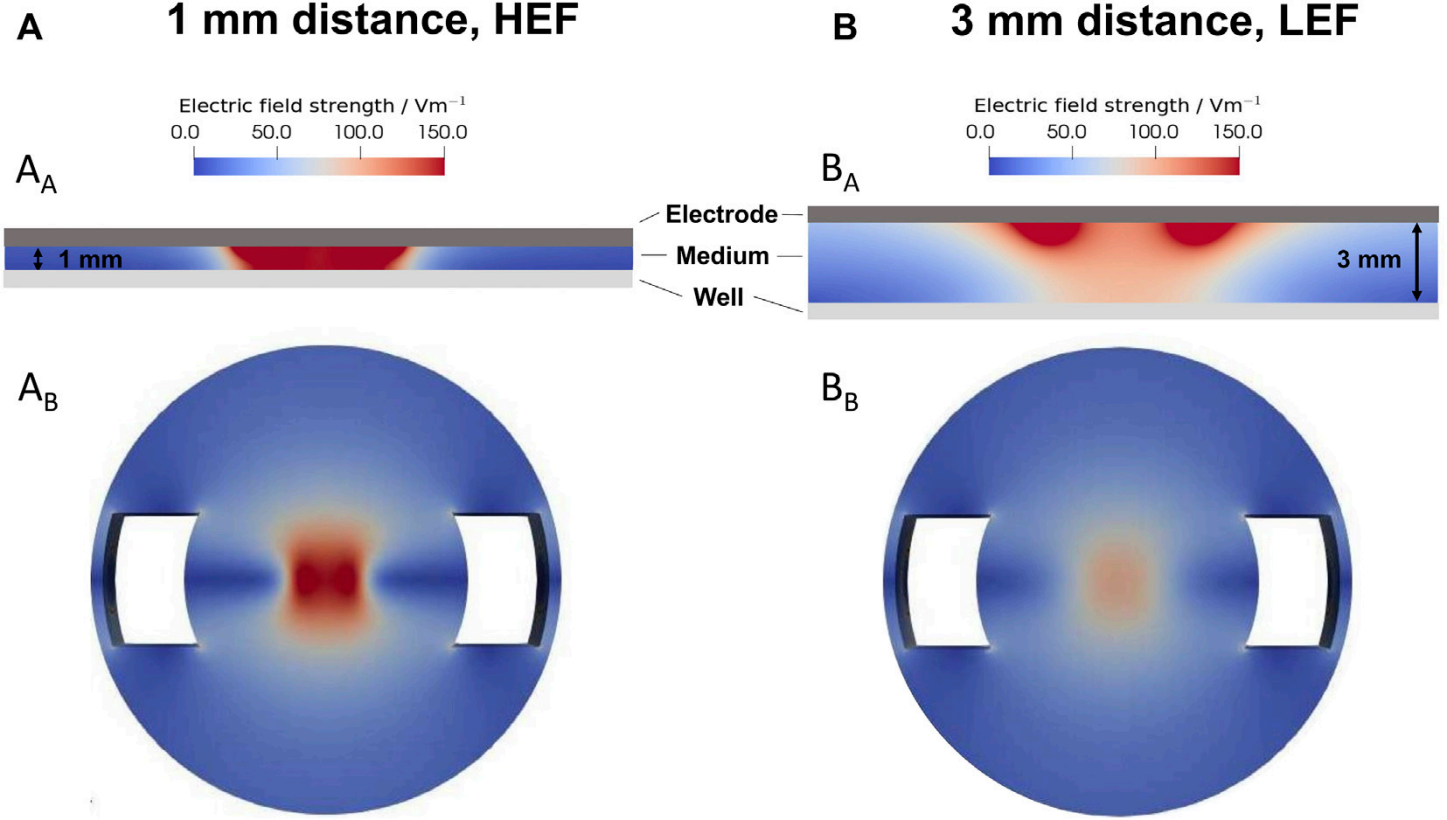
Simulation of the electric field using a sinusoidal signal with 20 Hz and 0.7 V_rms_. **(A)** Distribution of the electric field with a distance of 1 mm, leading to a higher electric field (HEF) **(B)** Distribution of the electric field with a distance of 3 mm, resulting in a lower electric field (LEF). A_A_ and B_A_ distribution of the electric field between the electrode and the well’s bottom. A_B_ and B_B_ electric field distribution on the well’s bottom. The 1 mm distance results in a higher electric field up to 150 V/m, and the 3 mm distance results in a lower electric field up to 100 V/m.

The electric field strengths were simulated for the 1 mm distance (Figure 2A) and the 3 mm distance (Figure 2B) from the electrode to the well’s bottom. The smaller gap results in a HEF with up to 150 V/m in the cell medium (Figure 2A_A_) and at the well’s bottom (Figure 2A_B_). The larger distance results in a LEF. The electric field strength decreased from approx 150 V/m near the electrode with increasing distance in the medium (Figure 2B_A_). According to the simulation, the electric field on the bottom of the well was a maximum of 100 V/m (Fig. B_B_).

The predicted total current through the well was 17.76 mA for the HEF. The current for the LEF was about 5% smaller. In preliminary current measurements, we recorded a current of about 0.77 mA. The measured smaller current can be mainly explained by the impedance of the electrode-electrolyte interface, which we did not consider in our model. Regarding the ratio between the measured and the predicted current, the prevailing electric field in the well could be about 20 times smaller than predicted by the simulations.

### 3.2 Cell viability

**FIGURE 3:**
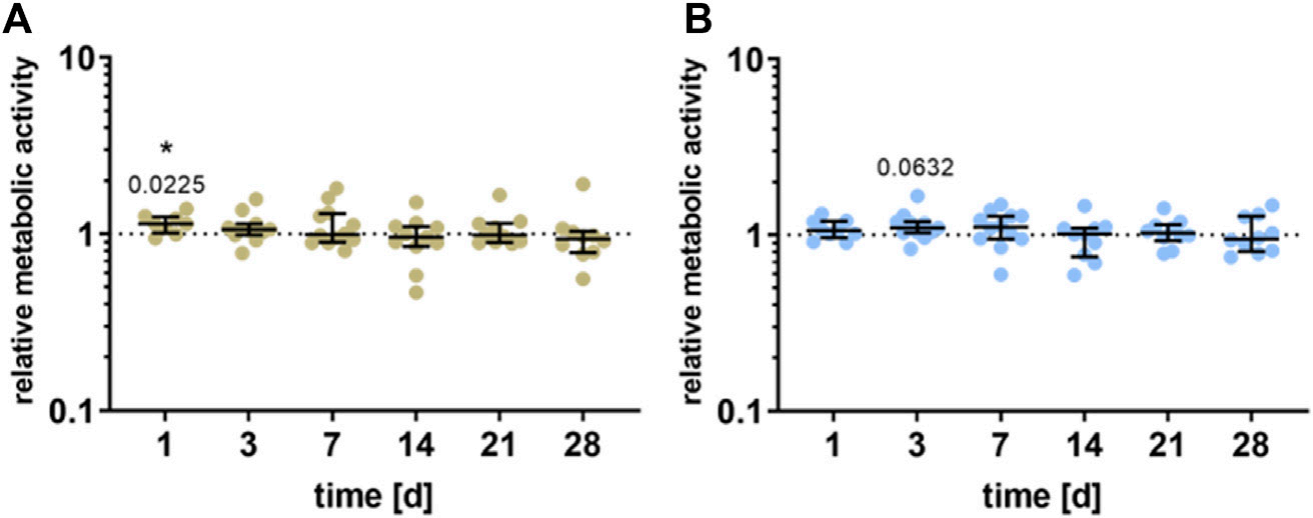
Metabolic activity of human pre-osteoblasts undergoing electrical stimulation. Pre-osteoblasts were stimulated over 31 days with two different electric fields [**(A)** higher electric field: HEF and **(B)** lower electric field: LEF] and without electrical stimulation (control). Analysis time points were 1, 3, 7, 14, 21, 28, and 31 days with assays performed 20 h after the last stimulation interval started. Metabolic activity of stimulated pre-osteoblasts related to the unstimulated control, determined *via* WST-1 assay. Results are shown as individual values with median and the 25%- and 75%-quartile to present the distribution of the total results [*n* ≥ 5]. *p < 0.05: significant differences between the stimulated and control groups.

Compared to unstimulated cells, a significant increase in the cell viability of the pre-osteoblasts could be detected 1 day after stimulation for the HEF (*p* = 0.0225) and a slight increase after 3 days for the LEF (*p* = 0.0632). Further stimulation time did not influence the viability. Besides, no significant difference between the electric fields could be detected (Figure 3).

### 3.3 Gene expression

**FIGURE 4:**
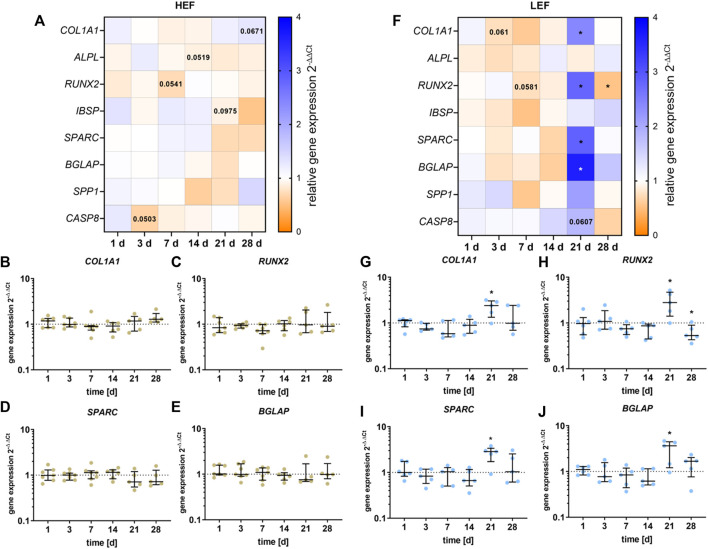
Gene expression analysis of osteogenic differentiation marker following stimulation with two different electric field strengths [**(A–E)** higher electric field: HEF, **(F–J)** lower electric field: LEF] related to the unstimulated control. Human pre-osteoblasts were stimulated over 28 days; the analysis time points were 1, 3, 7, 14, 21, and 28 days with assays performed 20 h after the last stimulation interval started. Gene expression rates were acquired *via* qPCR and related to the control using the 2^-∆∆Ct^ method. **(A,F)** Results within heatmaps are shown as medians whereby the downregulation is identified in orange [< 1], the upregulation in blue [> 1], and a similar gene expression as the control in white [ = 1]. **(B–E,G–J)** The distribution of the total results [*n* ≥ 5] are depicted as individual values with median and the 25%- and 75%-quartile. *p < 0.05: significant differences between the stimulated and control groups.

The gene expression of *COL1A1*, *ALPL*, *RUNX2*, *IBSP*, *SPARC, BGLAP, SPP1, RANKL,* and *CASP8* was analyzed following stimulation with HEF and LEF. The respective gene expression results are summarized in the heatmaps for HEF (Figure 4A) and LEF (Figure 4F). Since Ct values for *RANKL* did not reach the limit of 29, the results were not included in this study.

For cells stimulated with HEF, a slight downregulation for *RUNX2* after 7 days (*p* = 0.0541, Figure 4C) and for *ALPL* mRNA after 14 days (*p* = 0.0519, Supplementary Figure S3C) was detectable. After 21 days of stimulation, a slight reduction for *IBSP* could be detected (Supplementary Figure S3D) and after 28 days of stimulation, *COL1A1* (*p* = 0.0671) was upregulated (Figure 4B). Pre-osteoblasts stimulated with the HEF showed decreased *CASP8* gene expression (*p* = 0.0503, Supplementary Figure S3A) after 3 days of stimulation. No changes in the expression of *SPARC, BGLAP* and *SPP1* were observed due to the electric stimulation. (Figures 4D,E and Supplementary Figure S3B).

Stimulation with the LEF led to a downregulation of *COL1A1* mRNA on day 3 (*p* = 0.061), and of *RUNX2* on day 7 (*p* = 0.0581, Figures 4G,H). A stimulation over 21 days led to a significant upregulation of *COL1A1* (*p* = 0.0285), *RUNX2* (*p* = 0.034), *SPARC* (*p* = 0.0248), *BGLAP* (*p* = 0.0433) (Figures 4G–J), and a non-significant increase of *SPP1* (Supplementary Figure S3F). The upregulation did not continue until 28 days. Contrary, a significant downregulation of *RUNX2* (*p* = 0.0475) was detected. For cells stimulated with LEF, the mRNA transcription of *ALPL* and *IBSP* was not affected (Supplementary Figures S3G,H). Moreover, the *CASP8* gene expression was not influenced through the electric stimulation during the first days. At day 21 a slight increase in *CASP8* was detectable (*p* = 0.0607). (Supplementary Figure S3E).

### 3.4 Alkaline phosphatase activity and secretion of proteins

**FIGURE 5:**
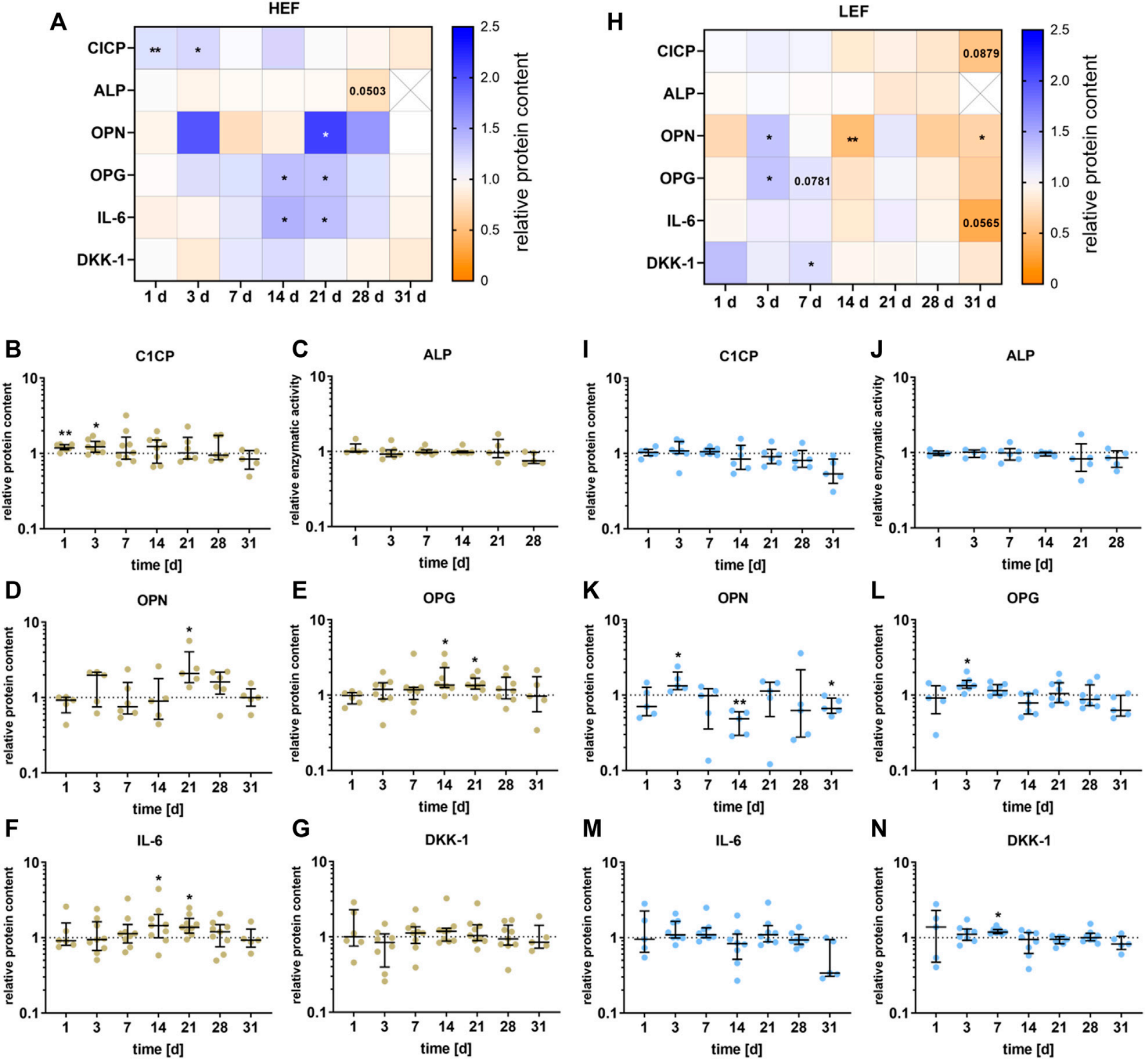
Secretion of proteins by pre-osteoblasts stimulated with two different electric field strengths [**(A–G)** higher electric field: HEF, **(H–N)** lower electric field: LEF] related to the unstimulated control. Pre-osteoblasts were stimulated over 31 days. The analysis time points were 1, 3, 7, 14, 21, 28 (for ALP), and 31 days (for CICP, OPN, OPG, IL-6, and DKK-1) with assays performed 20 h after the last stimulation interval started. Protein levels were acquired for CICP, OPN, OPG, IL-6, and DKK-1 from the supernatant and related to the total protein content of the supernatant. The activity level of ALP was generated through the lysis of the cells. The unstimulated control of each time point was used for normalization. **(A,H)** Results within heatmaps are shown as medians whereby the downregulation is identified in orange [< 1], the upregulation in blue [> 1], and a similar protein secretion as the control in white [ = 1]. **(B-G, I-N)** The distribution of the total results [*n* ≥ 5] are depicted as individual values with median and the 25%- and 75%-quartile. *p < 0.05, **p < 0.01: significant differences between the stimulated and control groups.

The release of different signaling proteins and the enzyme activity of ALP were analyzed for the two different distances from the electrode. The data are summarized in the heatmaps for HEF (Figure 5A) and LEF (Figure 5H). During the first stimulation days, CICP was significantly upregulated (day 1: *p* = 0.0034, day 3: *p* = 0.0104) following stimulation with HEF. A higher concentration could be detected for OPG and IL-6, with a peak at 14 and 21 and a decline at days 28 and 31. OPG and IL-6 were significantly upregulated during days 14 (OPG: *p* = 0.0156, IL-6: *p* = 0.0391) and 21 (OPG: *p* = 0.0158, IL-6: *p* = 0.0117). Moreover, OPN was significantly increased after 21 days (*p* = 0.0243) compared to the unstimulated control, and the concentration of OPG, IL-6, and DKK-1 dropped until 31 days. The electrical stimulation did not influence the ALP activity. Only after 28 days a slight decrease could be measured (*p* = 0.0503) (Figures 5B–G).

For the stimulation with the LEF, the CICP concentration was not significant influenced during the first days of stimulation but dropped until it reached its lowest value after 31 days (*p* = 0.0879) compared to the unstimulated control. ALP was not significantly influenced by the electrical stimulation but showed a similar downward trend at days 21 and 28 as CICP. The concentration of OPN and OPG was mainly upregulated 3 days (OPN: *p* = 0.0427, OPG *p* = 0.0156) and 7 days (OPG: *p* = 0.0781) after stimulation, and dropped after 14 days (OPN: *p* = 0.007). After 28 and 31 days a similar downturn as for CICP could be detected for OPN (*p* = 0.0368), OPG and IL-6 (*p* = 0.0565). DKK-1 was upregulated after 7 days (*p* = 0.0156) of electrical stimulation (Figures 5I–N).

### 3.5 Mineralization capacity

**FIGURE 6:**
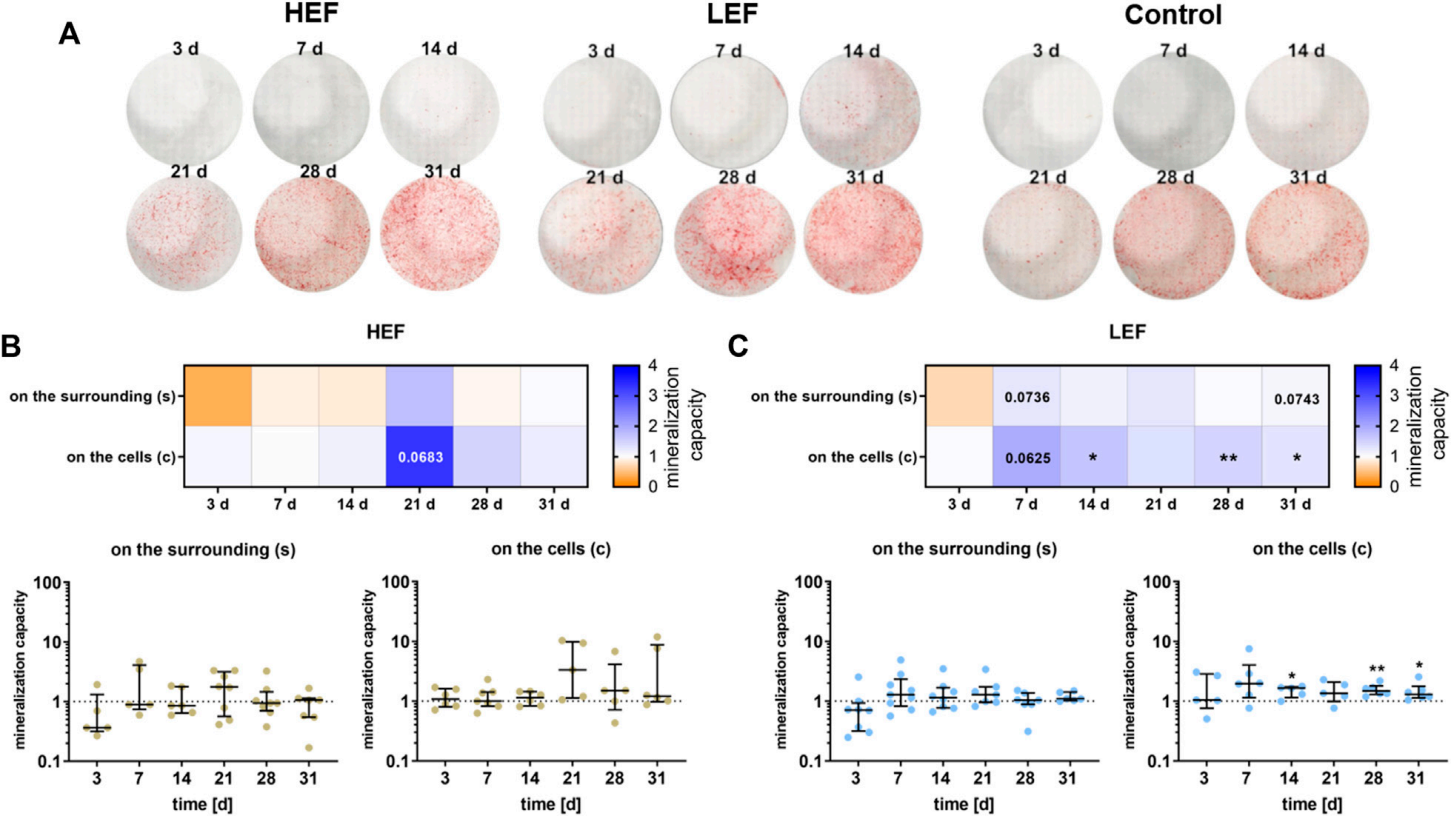
Mineralization behavior of human pre-osteoblasts undergoing electrical stimulation. Pre-osteoblasts were stimulated over 31 days with two different electric fields (higher electric field: HEF, lower electric field: LEF) and without electrical stimulation (control). Analysis time points were 3, 7, 14, 21, 28, and 31 days with assays performed 20 h after the last stimulation interval started. **(A)** Calcium nodule formation on the cells after electrical stimulation with HEF, LEF, and without electrical stimulation colored with alizarin red. **(B,C)** Evaluation of the mineralization behavior for HEF **(B)** and LEF **(C)** relative to the mineralization behavior of the unstimulated control. The amount of calcium nodule formation was determined after alizarin staining of the cells grown on coverslips (c) by proportioning the colored areas to the uncolored areas using ImageJ. The concentration of the calcium deposition on the surrounding (s) was determined after dissolving the mineralized matrix with HCl with the colorimetric Calcium Assay Kit. The unstimulated control of each time point was used for normalization. Results within heatmaps are shown as medians whereby the downregulation is identified in orange [< 1], the upregulation in blue [> 1], and a similar mineralization capacity as the control in white [ = 1]. The distribution of the total results [*n* ≥ 5] are depicted as individual values with median and the 25%- and 75%-quartile. *p < 0.05, **p < 0.01: significant differences between the stimulated and control groups.

The mineralization capacity of the human pre-osteoblastic cells under electrical stimulation was determined by the amount of calcium deposited on the surrounding well (Supplementary Figure S1) and the calcium nodule formation on the cells (Figure 6A). Deposition of mineralized matrix increased during cultivation time, both on the cells and the surrounding (Figures 6A–C). Stimulation without cells did not lead to calcium deposition (Supplementary Figures S2A,B). Cells cultivated under HEF showed an upregulation in the calcium nodule formation on the cells after 21 days (*p* = 0.0683) compared to unstimulated controls whereas calcium deposition on the surrounding well was not influenced (Figure 6B). Stimulation with the LEF led to higher precipitation of calcium on the surrounding on days 7 (*p* = 0.0736) and 31 (*p* = 0.0743). The calcium nodule formation was upregulated on day 7 (*p* = 0.0625) and significantly upregulated on days 14 (*p* = 0.0260), 28 (*p* = 0.005), and 31 (*p* = 0.0469) (Figure 6C).

### 3.6 Transcriptome analysis

Based on the probe intensity in the microarray data, the fold change of each gene transcript between unstimulated control cells and cells with HEF stimulation was estimated after 7 and 28 days, respectively. For the subsequent analysis only, transcripts were considered that have a higher/lower fold change value than 1/-1 (log2), which corresponds to double/halve expression values when comparing control and stimulation.

**FIGURE 7:**
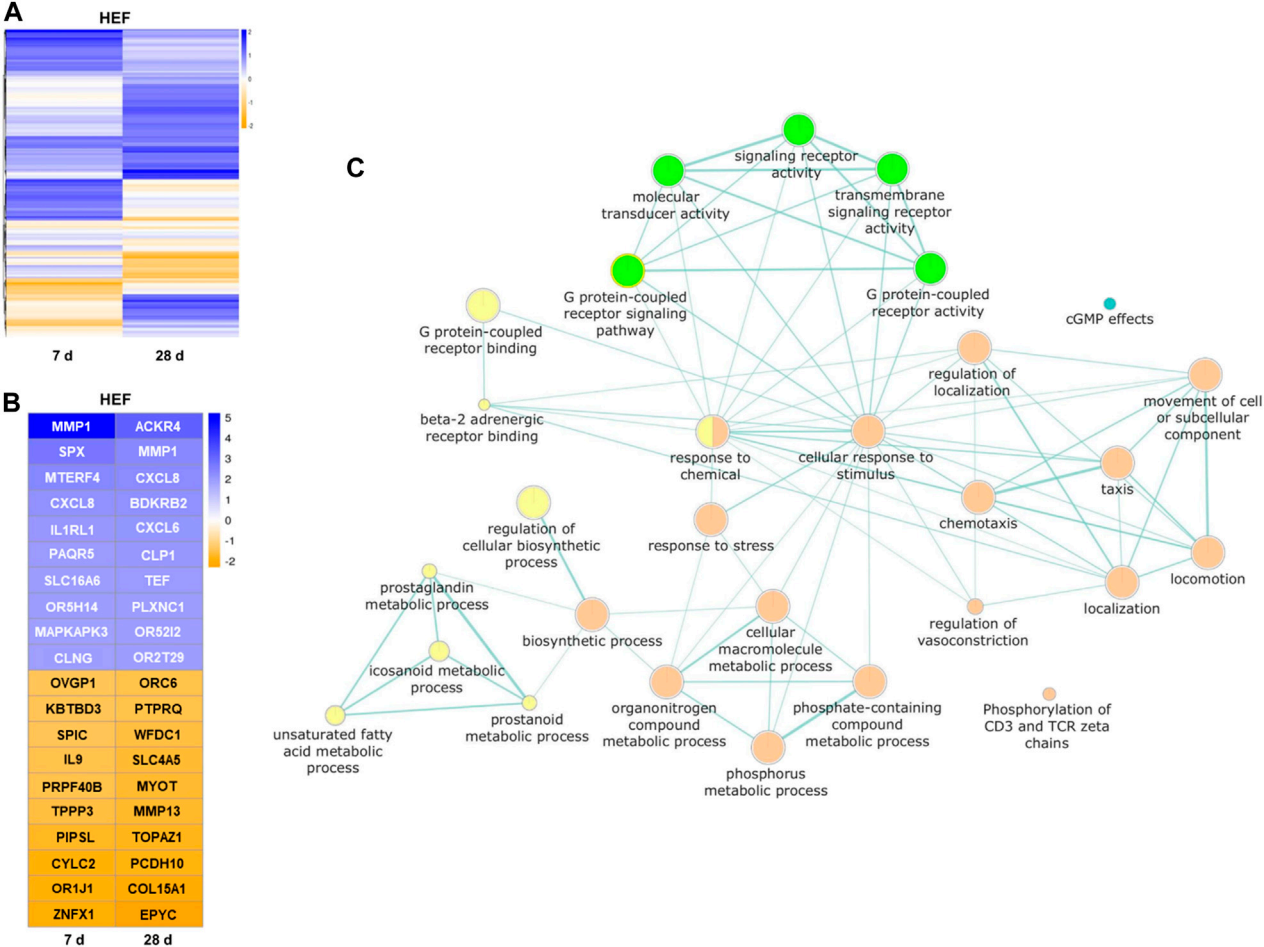
Results of the transcriptome analysis. **(A)** Heatmap of all genes with higher/lower fold change value than 1/-1 (log2), compared between unstimulated control cells and HEF stimulated cells after 7 and 28 days. **(B)** Heatmap of top 10 genes with highest and lowest fold changes (log2) each day. **(C)** Pathways influenced by the electric stimulation. Each node (circle) represents a distinct molecular or biological function, and edges (green lines) represent the number of overlapping genes, determined using a similarity coefficient (Shannon et al., 2003). Yellow and orange circles represent upregulated molecular or biological functions after 7 (yellow) and 28 days (orange) of HEF stimulation, respectively. Green and blue circles represent downregulated molecular or biological functions after 7 (green) and 28 days (blue) of HEF stimulation, respectively.

Interestingly, for most transcripts affected by the stimulation, the expression fold change is clearly distinct for both stimulation times (see Figure 7A). Only for a small set of genes stimulation with HEF induced an up- or downregulation at both time points. The remaining transcripts either show an up or down-regulated expression after 7 or 28 days of stimulation compared to unstimulated control, but not at both time points. Therefore, the gene expression at day 7 and 28 was considered separately in the pathway enrichment analysis.

The top 10 genes with highest and lowest expression fold changes are shown in Figure 7B. Among other genes, MMP1 and CXCL8 were both upregulated after 7 and 28 days of stimulation. SPX was one of the genes with the highest fold change for 7 days and ACKR4 for 28 days. Different G protein-coupled receptors were up- or downregulated e.g., OR5H14, OR52I2, OR2T29, OR1J1, and ORC6.

To gain an overview over the different cell responses revealed through the transcriptome analysis, a pathway enrichment analysis was created with separate lists for day 7 and day 28 containing all genes with distinct expression fold change (i.e., higher/lower fold change value than 1/-1 (log2)). Both gene lists were separately used as input for pathway enrichment analysis and to create gene enrichment maps with the help of g:profiler web-service. The resulting network shown in Figure 7C combines both gene enrichment maps of day 7 and day 28. It depicts all GO terms of molecular and biological functions that were overrepresented in the provided gene lists and further provides color-coded information, whether the associated genes were down- or upregulated. The network underlines the activation of different pathways by varying time points. Seven days stimulation led to a regulation of the binding, signaling pathway and activity of G protein-coupled receptors. Further, the metabolic processes of prostaglandin, eicosanoid, unsaturated fatty acids and prostanoid were upregulated. The stimulation over 28 days resulted in, among others, an increased cellular response to stimulus and stress, and different localization, locomotion reactions as well as an increase in the movement of cell or subcellular component. (Figure 7C).

## 4 Discussion

Electrical stimulation is known to influence bone healing processes and increase bone formation *in vivo*. Bhavsar et al. compared animal and clinical studies and revealed a positive influence of electrical stimulation in 77% of the animal studies and 73% of the clinical ones (Bhavsar et al., 2020).

Despite this availability of studies, the underlying cellular processes are not yet fully understood. While there is a substantial amount of data on short-term electric field exposure studies (Supronowicz et al., 2001; Creecy et al., 2013; Bique et al., 2016), there is a lack of knowledge on the biological response in long-term *in vitro* studies using directly coupled alternating electrical fields. Therefore, the focus of this research was to stimulate human osteoblasts with alternating electric fields over a period of 31 days to investigate the differentiation and mineralization behavior of the cells at different time points. Due to the chosen setup, we were able to stimulate the cells with two different electric field strengths. In addition, we used a numerical simulation to obtain information about the distributions of the HEF and LEF electric fields used for stimulation. Our main findings in this study were: 1) High alternating electric fields (HEF) induced increased secretion of CICP, OPN, OPG, and IL-6 across different time points. 2) Low alternating electric fields (LEF) induced gene expression of important osteogenic markers at day 21. Moreover, LEF increased the amount of mineralized matrix already after 7 days of stimulation. This increased mineralization was observed throughout the stimulation period. Thus, it can be concluded that directly coupled low-level alternating electric fields promote bone mineralization *in vitro*.

During the first days of stimulation, the cell viability of pre-osteoblasts was slightly increased, and no negative long-term effect was noticable. Similar outcomes were observed by other studies using alternating electric fields with a rise in cell number after 1 day’s stimulation or an increase in proliferation after 2 days (Supronowicz et al., 2001; Sahm et al., 2020). Studies with other stimulation systems revealed an influence on the cell number or metabolic activity during the first days of stimulation and no further change in osteoblast-like cells’ proliferation up to 29 days (Hronik-Tupaj et al., 2011; Zhu et al., 2017, 2019; Leppik et al., 2018; Bloise et al., 2020; Konstantinou et al., 2020; de Sousa et al., 2021). Even though these studies used different culture conditions, various stimulation systems and different cell types, a similar trend for the viability of pre-osteoblasts could be observed. As most of the studies analyzed the proliferation or viability, effects on cell apoptosis were not studied. *CASP8* is a widely described apoptotic marker for different cell types (Nicholson, 1999). The downregulation of *CASP8* with HEF after 3 days matches the slightly increased viability during the first days of stimulation. However, the upregulation of *CASP8* at day 21 with LEF was not reflected in the viability. In contrary, at day 21 the gene expression of several differentiation markers was increased. *CASP8* is not only known for its role in the cell death signaling but also has been shown to be important for the differentiation of the macrophage lineage (Kang et al., 2004). Furthermore, in osteoblastic cells, it was found that a reduction of *CASP8* transcripts decreased the expression of the osteogenic genes *BGLAP* and *PHEX* (phosphate-regulating neutral endopeptidase, X-linked gene) (Kratochvílová et al., 2020). Therefore, it can be assumed that an increase in *CASP8* mRNA following stimulation with LEF led to an increase to the investigated differentiation factors (*BGLAP, SPARC, COL1A1*) in our study. However, further work is required to evaluate a direct correlation between these signal cascades.

Besides *CASP8,* stimulation with LEF induced the mRNA transcription of *COL1A1*, *RUNX2*, *SPARC*, *BGLAP,* and *SPP1* after 21 days. These genes are involved in the induction of bone matrix formation and differentiation of osteoblasts (Bruderer et al., 2014). RUNX2 is one of the initial markers for osteogenic differentiation and decisive for the progression of pre-osteoblasts into active osteoblasts. During this differentiation process, RUNX2 is essential for the expression of bone matrix proteins like collagen 1, osteopontin, osteocalcin, and osteonectin (Huang et al., 2007; Rucci, 2008; Bruderer et al., 2014). Accordingly to Supronowicz et al. (2001) the detectable overexpression after 21 days suggests a promoting effect of electric fields on the further development of pre-osteoblasts into mature osteoblasts and for the further formation of mineralized bone matrix (Supronowicz et al., 2001). Also other studies observed a similar upregulation of osteogenic gene expression but with earlier upregulations after 7, 14, or 21 days (Hronik-Tupaj et al., 2011; Creecy et al., 2013; Wechsler et al., 2016; Zhu et al., 2017). Different stimulation systems, applied frequencies, voltages, or even the cell type origin or cultivation conditions can trigger different gene expressions at different time points (Griffin et al., 2011; Bique et al., 2016; Leppik et al., 2020). Chaudhari et al. examined the *OPG* expression using a variety of frequencies and voltages and revealed up- and downregulations of *OPG* depending on the electric field strength (Chaudhari et al., 2021). This observation can be supported with our result obtained for the secretion of OPG by the pre-osteoblasts stimulated with different electric field strengths. Besides OPG, the HEF upregulated the protein synthesis rate of IL-6, OPN and DKK-1 after 14 or 21 days of stimulation. A similar trend was detected for the LEF at an earlier stage, as the upregulation started already after the first day of stimulation. The cells seem to react differently to the varying electric field strengths underlining the importance of an optimal electric field used for stimulating bone cells.

Moreover, LEF caused a significant upregulation of the mineralized matrix after 14, 28, and 31 days of stimulation. During these days, the amounts of the investigated proteins were not influenced or even downregulated, suggesting a promoting effect on the mineralization capacity. In particular, our data demonstrate the correlation of secreted OPN on the mineralization capacity of pre-osteoblasts following stimulation with LEF. OPN in its phosphorylated state inhibits mineralization processes (Jono et al., 2000), and as the amount of ALP did not vary during stimulation, phosphorylated OPN can be assumed. When comparing mineralization and OPN release, a coherent trend becomes apparent. Is OPN upregulated or similar to the control, the mineralization capacity is not upregulated. The amount of mineralized matrix increases significantly when OPN is downregulated, confirming the regulatory effect of OPN on mineralization during electric stimulation (Jono et al., 2000). The increased deposition of mineralized matrix was detectable not only on the cell-seeded coverslips but also outside of them in the surrounding wells. It is likely that secreted microvesicles, which include Ca^2+^ and P_i_ ions (Bourne et al., 2021), are circulated throughout the well. At those sites where type 1 collagen has been deposited, crystallization of CaP then occurs (Bourne et al., 2021). We assume that osteoblastic deposition of a collagen matrix is not only limited to the coverslips, so that a clear mineralization can also be detected in the complete well. Moreover, as described before, the regulation of the mineralization layer seems to be dependent on the alkaline phosphatase activity and OPN secretion.

HEF, on the contrary, did not affect the mineralization processes to the same extent as LEF did, but led to a prolonged and later upregulation of secreted protein levels of OPG, IL-6, OPN, and DKK-1. These proteins are known to be involved in bone remodeling processes (Rucci, 2008; Einhorn and Gerstenfeld, 2015; Si et al., 2020). OPG is known for its influence on bone growth through the RANKL/RANK/OPG signaling system (Boyce and Xing, 2008). OPN can influence progenitor cells like mesenchymal stem cells, hematopoietic stem cells and osteoclast migration and adhesion (Walker et al., 2010; Si et al., 2020). A common underlying mechanism triggered by the electrical stimulation for DKK-1 and IL-6 may be the Wnt signaling pathway, as both are known to be involved in this pathway (Malysheva et al., 2016). DKK-1 is mainly known for inhibiting osteoblastic function but can also play a role in the mineralization processes of mature osteoblasts (Westendorf et al., 2004; van der Horst et al., 2005). IL-6 has a controversial role in bone remodeling processes as it can activate or deactivate osteoblasts and osteoclasts, probably depending on the presence of other cytokines and the differentiation stage of the cells (Blanchard et al., 2009; Feng et al., 2017). Because these proteins were upregulated in HEF over a longer stimulation time than in LEF, a stronger influence of HEF on bone remodeling and possibly on bone resorption processes can be assumed. Further studies are necessary to prove this assumption. The cultivation of osteoclast-like cells with the supernatants generated from stimulated osteoblasts or the simultaneous stimulations in co-cultures may give insights into the activation and differentiation of osteoclasts through the released cytokines. The interaction between osteoblast and osteoclast is important to understand the up- and downregulation of cytokines which are essential for promoting healing rates in stimulated bone. A 3D printed scaffold can generate a surrounding to study the crosstalk between different cells like osteoblasts, osteoclast or endothelial cells (Sieberath et al., 2020; Kanwar and Vijayavenkataraman, 2021). It would allow the differentiation of osteoblasts into osteocytes, as 3D systems are necessary for generating osteocytes *in vitro* (Sawa et al., 2019). Further, a 3D system would enable better comparison between *in vitro* and *in vivo* conditions to increase knowledge about the influence of electrical stimulation on bone remodeling processes. Future *in vivo* studies are necessary to confirm the assumptions made through this study and to examine crosstalk between different cell types. One conceivable application would be the insertion of an electrically active stimulation device into an artificial hip stem or the use in bone defects of critical size (Zimmermann U et al., 2021). As *in vivo* processes are more complex through the interplay of different cells, the fluid flow and the bone matrix, it can be that the observed effects are diminished, unchanged or intensified. *In vivo* analysis of the healing process under electrical stimulation using sensors like the bioMEMS are possible to generate more data about the optimal stimulation conditions for increasing bone healing rates under electric stimulation *in vivo* (McGilvray et al., 2015).

To identify fundamental key factors and signaling pathways, which are the link between the external field and the increased differentiation, further studies are needed. The calcium-sensing receptor and channels like piezo 1 and 2 seems to be key factors which might be involved in the signal transduction (Lee et al., 2014; Cianferotti et al., 2015). A first try to reveal the underlying signaling cascades was done by the transcriptome analysis of cells stimulated 7 and 28 days with the HEF. The comparison between the early and late time point emphasizes the importance of a long-time stimulation *in vitro* as different gene expression profiles were observed. Under electric stimulation, the gene expression changed depending on the stimulation time. The gene expression profile of the 7 day stimulation revealed an increase in the metabolic processes of eicosanoids like prostaglandin and prostanoid and a reduction of the G protein-coupled receptor (GPCR) activity. Eicosanoids are known to be important signaling molecules and are mainly recognized by cell membrane GPCRs (Calder, 2020). As the GPCR activity was reduced, the enhanced eicosanoids processes might suggest an increase in the cell communication with other cell types triggered through the electric stimulation. Another possible target could be nuclear receptors in the cells like the peroxisome proliferator-activated receptors which are known to be involved in proliferation and differentiation processes (Chinetti et al., 2000). The 28 days stimulation led to an increase in cellular responses to stimulus, stress and to an activation and regulation of locomotion, localization and movement. The influence of electrical stimulation in the movement and migration of the cells is mainly known for direct current and electromagnetic field stimulation (Ferrier et al., 1986; Mycielska and Djamgoz, 2004; Zhang et al., 2018). As the influence of alternating electric fields on the migration was not yet described, further research needs to be done regarding long-term stimulation with alternating fields and the observation of the movement of the cell or subcellular components. In the conjunction with the enhanced migration, the transcriptome analysis revealed increased chemotaxis and taxis. It can be assumed that released chemokines influence the migration behavior not only for the osteoblastic cells but also for other cells types. The increased gene expression of osteoblastic differentiation factors at day 21 might be the possible trigger for further chemotaxis and migration. These observations underline the importance of co-culture models to understand the interplay between different cell types. The implemented transcriptome analysis is, through the pooling of analyzed samples, just a first insight. Further investigations regarding the gene expression profiles are necessary. More time points can give a better understanding on how the electric stimulation is changing the signaling cascades over time. Analysis from cells 24 h after the start of the electric stimulation might reveal cascades which are involved in the proliferation. Advanced timings are important to understand cell signaling which is fundamental for the increased mineralization.

In order to achieve more comprehensive comparability between studies, the specification of the electric field and validation of the utilized fields should be sought after. With information about the applied electric fields, research data can be put in the proper context, and the optimal electric field for bone regeneration may be identified. The numerical simulation of the electric fields used in our study is the first attempt to estimate the field resulting from the used parameters. One limitation of the numerical simulations is the assumption that the electrode-electrolyte interface impedance does not affect the current density distribution on the electrode surface. At low frequencies such as 20 Hz, an electrochemical double layer may arise on the electrode, reducing the electric field in the surrounding. Moreover, the model makes the postulation of an electrochemically inert and stable system. Due to the cells, the progressive mineralization processes, and the electric field, electrochemical reactions in the system might occur. These electrochemical reactions would influence the electric field as well. Furthermore, electrochemical reactions on the electrode could lead to corrosion on the surface of the electrode, resulting in reduced field strengths. The first steps in addressing these issues have been made and will be refined in future research (Zimmermann J et al., 2021). Measurements with electrochemical impedance spectroscopy, local measurements of the induced voltage in the medium, and the constant documentation of the current and the voltage during stimulation will give more information about the electric field strength and possible confounding factors. Investigations about probable corrosion processes or deposits on the surface of the Ti6Al4V during electrical stimulation could answer if electrochemical reactions on the electrode arise and how strongly they influence the electric field strength. This is also important when thinking about future *in vivo* applications. *In vivo* devices may need to be controllable as the electric field is reduced by the deposition of extracellular matrix. With an adjustable device, a desired electric field can be kept constant over time by increasing parameters such as frequency or voltage. Despite a presumed reduction of the simulated field strengths during stimulation in this study, the percentage difference between the fields will remain the same. Thus, it can be assumed that the two electrode configurations with different distances will always lead to different electric fields with constant ratios.

## 5 Conclusion

In conclusion, our study demonstrates the importance of long-term stimulation for a better understanding of the effects of electric fields on osteoblastic cells. The impact of electrical stimulation on the cells can change as time progresses, so experiments on long-term stimulation are essential. The metabolic activity was promoted during the first days of stimulation, gene expression and protein release changed over time. The electrical stimulation with low frequency alternating electric fields activated the osteogenic differentiation of pre-osteoblast and influenced bone remodeling processes. The LEF led to an increase in the mineralization capacity over time until 31 days. In contrast, HEF did not influence the mineralization but led to a later but longer-lasting increase in the bone remodeling markers OPN, OPG, IL-6, and DKK-1. This could predict a higher efficiency in bone formations due to lower electric fields. Further experiments with immune cells and osteoclasts should be performed to understand better the influence of the released cytokines and the crosstalk between different cell types and the resulting bone remodeling processes under long-term electrical stimulation. With increased studies of electric field strengths *in vitro* and *in vivo* through validation and simulation of field distribution, more information on optimal stimulation parameters can be obtained.

## Data Availability

The datasets generated during the current study are available from the corresponding author on reasonable request.
