# Supplementary material for: Long-term stimulation with alternating electric fields modulates the differentiation and mineralization of human pre-osteoblasts
Source: Front Physiol. 2022 Sep 30;13:965181. doi: 10.3389/fphys.2022.965181 (PMC9562827; doi:10.3389/fphys.2022.965181)
Supplement: Supplementary file 1 [file DataSheet1.docx]

Supplementary Material


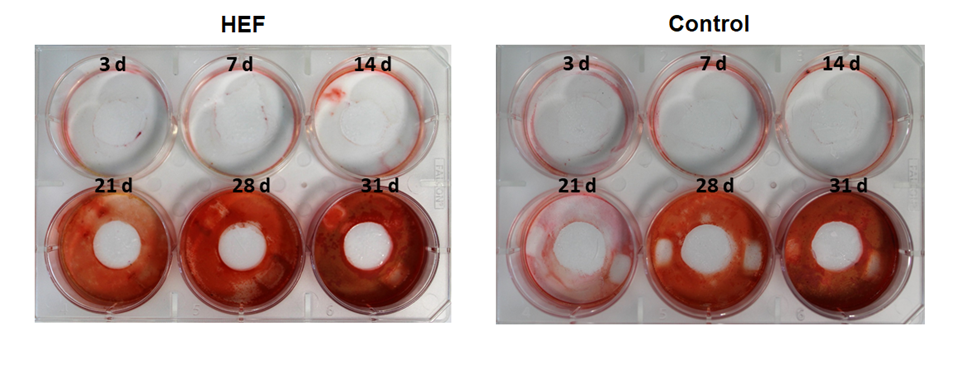


Supplementary Figure 1: Example of calcium deposition in the well plate during stimulation of osteoblasts over 31 days for the HEF and the control with not connected electrodes. The recess in the center represents the position of the cell-populated coverslip, which was removed before coloring with alizarin red.


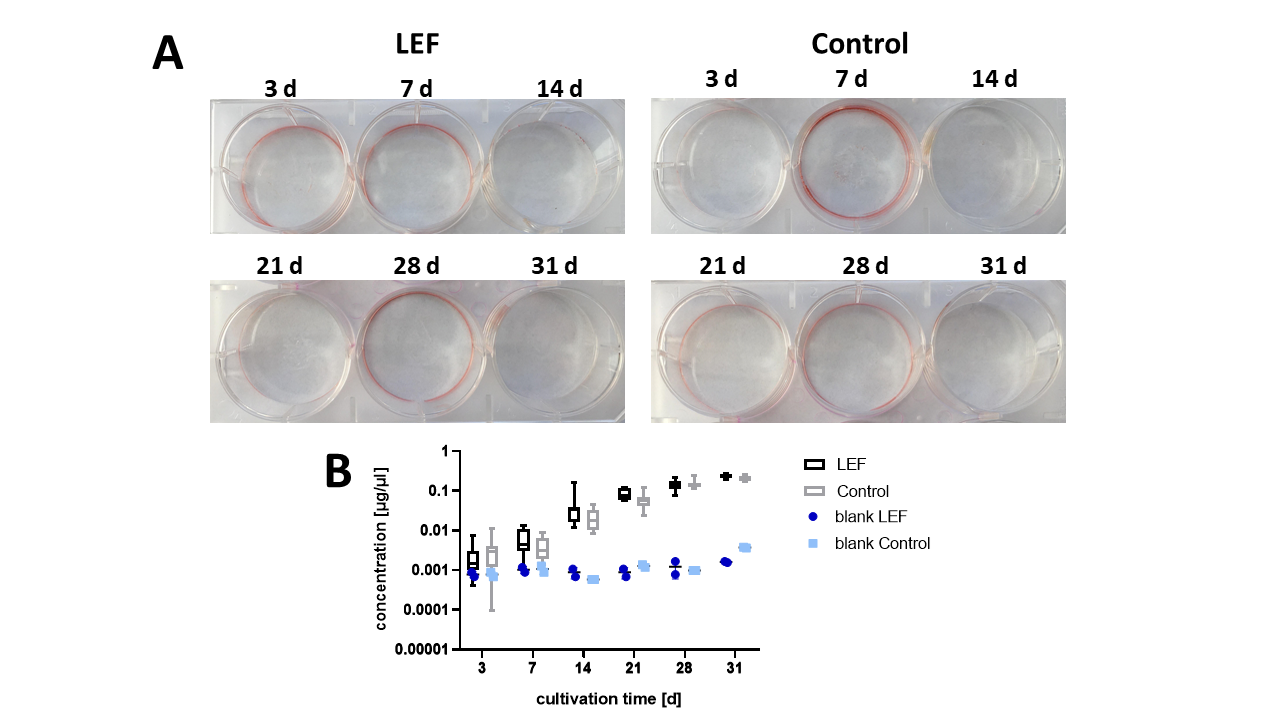


Supplementary Figure 2: Example of calcium deposition in the well plate during stimulation with and without osteoblasts for LEF and control over 31 days. (A) Well bottom colored with alizarin red after stimulation without cells. (B) The concentration of the calcium deposition on the surrounding determined with the colorimetric Calcium Assay Kit after dissolving the mineralized matrix with HCl. The calcium deposition after stimulating osteoblasts with LEF is shown in black, for the control when osteoblasts were not stimulated in grey (n≥5). The calcium deposition without cells is shown in blue, the blank for LEF without osteoblasts in dark blue, the blank for the control without cells and stimulation in light blue (n=2).


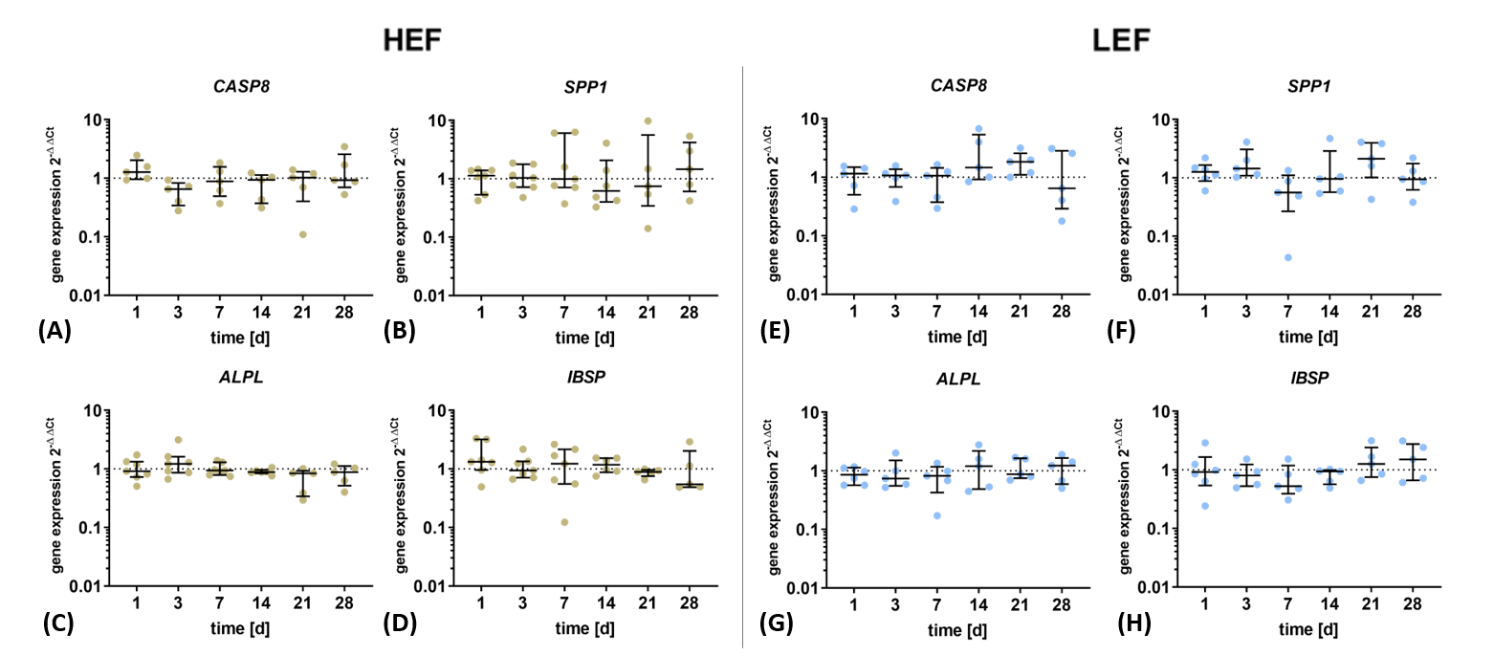


**Supplementary Figure 3:** Gene expression analysis of caspase 8 (*CASP8*), osteopontin (*SPP1*), alkaline phosphatase (*ALPL*) and integrin binding sialoprotein (*IBSP*) following stimulation with two different electric field strengths [(A-D) higher electric field: HEF, (E-H) lower electric field: LEF] related to the unstimulated control. Human pre-osteoblasts were stimulated over 28 days; the analysis time points were 1, 3, 7, 14, 21, and 28 days with assays performed 20 h after the last stimulation interval started. Gene expression rates were acquired via qPCR and related to the control using the 2^-∆∆Ct^ method. The distribution of the total results [n ≥ 5] are depicted as individual values with median and the 25%- and 75%-quartile**.**
